# Novel ADCs and Strategies to Overcome Resistance to Anti-HER2 ADCs

**DOI:** 10.3390/cancers14010154

**Published:** 2021-12-29

**Authors:** Elena Díaz-Rodríguez, Lucía Gandullo-Sánchez, Alberto Ocaña, Atanasio Pandiella

**Affiliations:** 1 Instituto de Biología Molecular y Celular del Cáncer, CSIC-IBSAL and CIBERONC, 37007 Salamanca, Spain; ediaz@usal.es (E.D.-R.); lgandullo@usal.es (L.G.-S.); 2 Departamento de Bioquímica y Biología Molecular, University of Salamanca, 37007 Salamanca, Spain; 3 Hospital Clínico San Carlos, Centro de Investigación Biomédica en Red de Oncología (CIBERONC), 28040 Madrid, Spain; alberto.ocana@salud.madrid.org

**Keywords:** HER2, ADC, drug resistance

## Abstract

**Simple Summary:**

A proportion of breast tumors bear the oncogenic transmembrane tyrosine kinase protein HER2. Even though therapies that target HER2 have changed the prognosis and quality of life of patients with HER2+ breast tumors, resistance to those therapies is still an important clinical problem. To improve the management of those tumors, a new category of antitumor agents, antibody-drug conjugates (ADCs), has emerged. These agents are created by linking a potent cytotoxic to an antibody that recognizes a membrane protein. Two anti-HER2 ADCs have been approved by the FDA for clinical use and several others are under development. The structure, mechanism of action, and resistance mechanisms to ADCs are reviewed in the present work, as well as potential strategies to overcome resistance to clinically approved anti-HER2 ADCs, including novel anti-HER2 ADCs.

**Abstract:**

During recent years, a number of new compounds against HER2 have reached clinics, improving the prognosis and quality of life of HER2-positive breast cancer patients. Nonetheless, resistance to standard-of-care drugs has motivated the development of novel agents, such as new antibody-drug conjugates (ADCs). The latter are a group of drugs that benefit from the potency of cytotoxic agents whose action is specifically guided to the tumor by the target-specific antibody. Two anti-HER2 ADCs have reached the clinic: trastuzumab-emtansine and, more recently, trastuzumab-deruxtecan. In addition, several other HER2-targeted ADCs are in preclinical or clinical development, some of them with promising signs of activity. In the present review, the structure, mechanism of action, and potential resistance to all these ADCs will be described. Specific attention will be given to discussing novel strategies to circumvent resistance to ADCs.

## 1. Introduction

HER2 belongs to the ErbB/HER family of transmembrane tyrosine kinases, which also includes EGFR, HER3, and HER4 [1,2]. Increased activity of HER2 has been linked to the initiation and progression of different tumors [3]. In fact, elevated levels of HER2 were initially identified in a subset of breast tumors, and such overexpression correlated with poor prognosis [4]. That fact prompted the development of targeted therapies specifically designed to act on HER2 [2]. One of these successful therapies relies on the use of antibody-drug conjugates (ADCs) [5,6]. In this review, we will discuss currently approved anti-HER2 ADCs and strategies to overcome resistance against them.

## 2. ADCs’ General Structure

**Figure 1:**
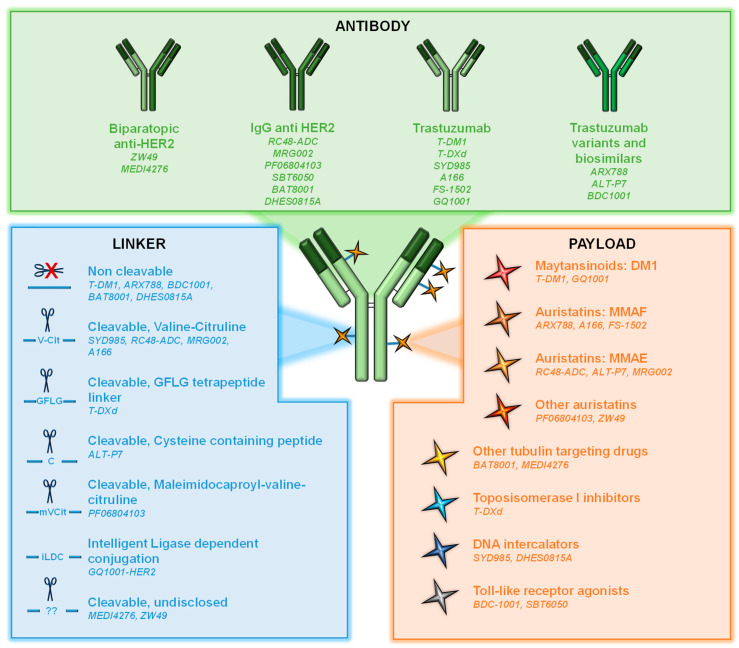
Schematic representation of an ADC. The three basic components of an ADC are shown: antibody, payload, and linker. In addition, different alternatives of those components in anti-HER2 ADCs are illustrated.

ADCs are sophisticated versions of antibodies that target cell surface proteins [7,8]. They consist of three components: (i) an antibody directed to a cell surface protein; (ii) a cytotoxic agent (also known as payload or warhead); and (iii) a linker used to attach the cytotoxic to the antibody (Figure 1).

At the time of writing this review, twelve ADCs have been approved by the FDA and several hundred are now being investigated in clinical or preclinical settings. In the case of the anti-HER2 ADCs, most of them use, as an antibody, the trastuzumab backbone or a modified version of this antibody (e.g., ARX788).

**Figure 2:**
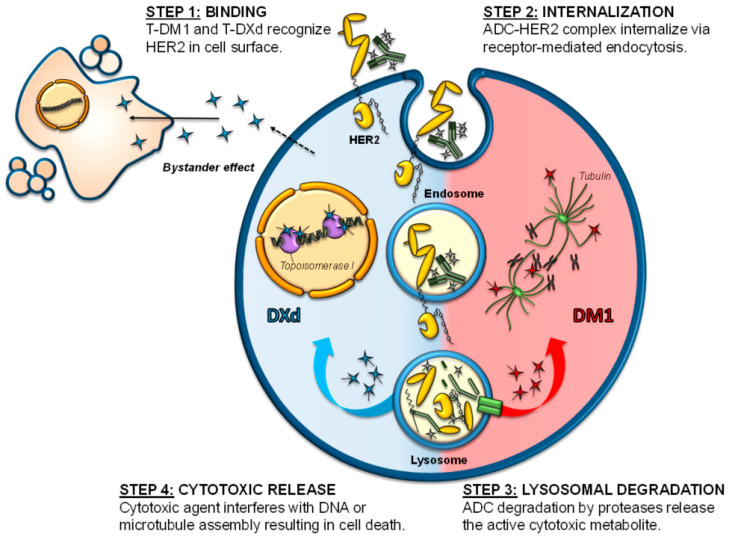
Mechanism of action of anti-HER2 ADCs. The figure schematizes the different steps involved in the action of anti-HER2 ADCs, which include: (**step 1**) binding of the ADC to HER2, (**step 2**) internalization of the ADC-HER2 complex, (**step 3**) lysosomal degradation of the ADC, and (**step 4**) release of the payloads to the cytosolic compartment. DM1 inhibits microtubule polymerization by targeting tubulin, while DXd moves to the nucleus causing DNA damage by targeting topoisomerase I.

**Cytotoxic agents** used as payloads in ADCs can be divided into two groups: antimicrotubule compounds (maytansinoids or auristatins) and DNA-damaging agents (topoisomerase inhibitors or DNA intercalators). *Maytansinoids* as mertansine (DM1) or DM4 are synthetic derivatives of maytansine, an inhibitor of microtubule polymerization. They bind to tubulin, leading to mitotic arrest and cell death [9,10] (Figure 2). DM1 is the warhead of trastuzumab-emtansine (T-DM1), the first ADC approved for the treatment of solid tumors [11]. *Auristatins* such as monomethyl auristatin E (MMAE) and F (MMAF) are synthetic compounds derived from the natural antimitotic drug dolastatin 10, isolated from the marine sea hare *Dolabella auricularia* [12,13].

*Topoisomerase I inhibitors* include deruxtecan (DXd), the warhead used in the recently approved anti-HER2 ADC trastuzumab-deruxtecan. DXd is a highly potent derivative of exatecan, a water-soluble structural analog of camptothecin with unique hexacyclic structure [14,15]. These agents bind and stabilize the topoisomerase I-DNA complex, inhibiting the religation of DNA breaks and thereby hampering DNA replication and triggering apoptotic cell death (Figure 2). *DNA intercalators* act on the minor groove of the DNA, inducing double-strand breaks and subsequent cell apoptosis [16]. An example of these payloads is duocarmycin, which has been incorporated, for example, in the development of trastuzumab-duocarmycin or SYD985. In this case, as duocarmycin is rapidly degraded in plasma, it is incorporated into the ADC in its inactive pro-drug form, seco-duocarmycin-hydroxybenzamide-azaindole (seco-DUBA). Once released into the cells, duocarmycin binds the minor groove of the DNA in an AT-rich region, causing DNA alkylation and DNA damage in both dividing and non-dividing cells [16,17].

An important aspect to be considered in the generation of ADCs is the amount of cytotoxic moiety loaded on the antibody, also known as DAR (Drug to Antibody Ratio). In fact, different DARs have been used for the development of different ADCs, with the most typical DAR being between 2 to 8 [7,18] (Table 1).

The **linkers** used to attach the payload to the antibody can be classified into cleavable and non-cleavable (Figure 1) [19,20]. The specific design of the linkers determines many aspects of ADC pharmacology, such as drug stability into circulation, tumor cell permeability, or DAR [21,22]. In *non-cleavable linkers*, the bond between the cytotoxic and the antibody is resistant to proteolytic degradation. These kinds of linkers exhibit higher stability at the expense of lower membrane permeability. In the case of *cleavable linkers*, these can be cut in response to environmental factors such as pH (acid-labile linkers as hydrazine linkers), specific lysosomal proteases as cathepsin B (protease-cleavable linkers), or glutathione reduction (disulfide linkers) [23]. These linkers can facilitate the release of the chemical agent within the microenvironment acting on tumoral cells in proximity that can express or not express the target. This action is called the bystander effect [24].

In addition to the chemical linker, the method leading to that binding is another important issue. Initially, conjugation relied on lysine and interchain cysteines [19], although currently most ADCs rely on interchain disulfide cysteines for conjugation, in which the interchain disulfide bonds are reduced by an excess of reducing agent, disrupting them and freeing sulfhydryl groups, finally resulting in more homogeneous ADCs and DAR numbers [25]. In addition, novel site-specific conjugation methods using unique linker chemistries leading to homogeneous ADCs with desired DARs have emerged [25,26], such as engineering unnatural aminoacids as *p*-acethylphenylalanine or *p*-azidomethyl-L-phenylalanine [27,28].

## 3. Mechanism of Action of ADCs

When ADCs bind to their target at the cell surface, the antigen-ADC complex is internalized via receptor-mediated endocytosis (clathrin- or caveolae- endocytosis) or pinocytosis [29,30] (Figure 2). Internalization results in inward budding of the cell membrane to form an early endosome that will further mature into a late endosome before fusing to lysosomes [31]. The whole antigen-ADC complex is then degraded. In the case of ADCs with cleavable linkers, cleavage can occur in early or late endosomes or even in the lysosomes. Nonetheless, in ADCs with non-cleavable linkers, cleavage happens at the lysosomal compartment and requires the action of acidic proteases such as cathepsin B or plasmin [31].

In both cases, and after cleavage, ADC payloads are small molecules that can be transported from the lysosomal lumen to the cytosol, where they are able to induce their action either in that compartment (e.g., antimicrotubular drugs) or after translocation to the nucleus (e.g., DNA damagers). In any case, the final consequence would be the induction of cell death [32]. In some cases, the cytotoxic payload, once liberated in the cytoplasm, may be expelled from the cell or be released in the tumor microenvironment after cell death. In that circumstance, cells that do not express the target antigen may be affected by the cytotoxic payload, inducing what is known as the bystander killing effect [24,33] (Figure 2). This effect depends on several factors, such as the kind of linker or the properties of the payload, but the presence of cell-permeable payloads will increase the effect [34].

## 4. ADCs in HER2-Positive Breast Cancer

### 4.1. Approved Anti-HER2 ADCs

#### 4.1.1. T-DM1

Trastuzumab emtansine, ado-trastuzumab emtansine, or T-DM1 (Kadcyla™), was the first ADC approved for the treatment of solid tumors, in February 2013 [11]. It bases its activity on the action of the anti-HER2 monoclonal antibody trastuzumab that is covalently bound through an uncleavable thioether linker to the cytotoxic agent mertansine (emtansine or DM1, Figure 1) [35,36]. In the case of T-DM1, the DAR is 3.5. All the cytotoxic functions of trastuzumab, such as antibody-dependent cell-mediated cytotoxicity (ADCC), cell cycle arrest, or signaling inhibition, are maintained and potentiated by the antitumoral actions of DM1 [36,37,38]. DM1 catabolites, once released into the cytosol, will inhibit microtubule polymerization in target cells, causing cell cycle arrest in G_2_/M [36], mitotic catastrophe, and cell death (Figure 2) [39,40].

T-DM1 has proved its value and effectiveness in different clinical settings, including early and advanced breast cancer. Thus, more than 100 clinical trials have evaluated or are studying different aspects of T-DM1 biology and function, either alone or in combination, on both primary tumors and metastatic lesions [35]. Approval of this agent in metastatic breast cancer was based in two phase III clinical trials, EMILIA and TH3RESA, that demonstrated significant improvement in progression-free survival (PFS) and overall survival (OS) with a good safety profile. The EMILIA trial compared the action of T-DM1 with that of the anti-HER2 therapy lapatinib, in combination with capecitabine, and after progression to a trastuzumab-based first line therapy [11]. The TH3RESA trial compared T-DM1 with the physician’s choice in a heavily pretreated population [41]. More recently, the KATHERINE trial allowed the approval of T-DM1 in the post-neoadjuvant setting for patients with residual invasive breast cancer after neoadjuvant therapy [42]. MARIANNE was the first phase III trial to evaluate the efficacy of T-DM1 in first line treatment. Surprisingly, the addition of pertuzumab, another anti-HER2 antibody approved in the clinic, to T-DM1 did not improve the trastuzumab plus taxane arm in terms of OS, PFS or overall response rate (ORR), although the safety profile and tolerability were much better [43,44]. Thus, in summary, T-DM1 is now indicated as a single agent in the adjuvant treatment of patients with HER2-positive (HER2+) early breast cancer who have residual invasive disease after neoadjuvant taxane and trastuzumab-based treatment, and in patients with metastatic breast cancer who previously received trastuzumab and/or a taxane, and either received previous therapy for metastatic disease or developed disease recurrence during or within six months of completing adjuvant therapy.

In general, T-DM1 is associated with manageable (grade 1/2) adverse events, such as gastrointestinal toxicity, neuropathy, left ventricular ejection fraction decline, or, in a minority of cases, high grade toxicities such as thrombocytopenia and increase in liver enzymes [45,46].

#### 4.1.2. Trastuzumab Deruxtecan

In December 2019, the trastuzumab-based ADC trastuzumab-deruxtecan (T-DXd, Enhertu™) reached oncology clinics. T-DXd was initially named DS8201a and has been approved for the management of advanced stage HER2+ breast cancer. As with T-DM1, the antibody backbone is trastuzumab, but in this case combined to the topoisomerase I inhibitor deruxtecan, with a final DAR of 7–8 [47]. A cleavable tetrapeptide linker (Gly-Phe-Leu-Gly, GFLG, Figure 1), potentially degradable by the lysosomal proteases cathepsin B and L, is used. DXd, once released, will bind to the topoisomerase I-DNA complex through hydrogen bonds resulting in a ternary stable complex that will prevent DNA religation, generating single and double-strand breaks and leading to apoptotic cell death (Figure 2) [47]. In addition, the liberated payload will reach high intracellular concentration and is membrane permeable [47], therefore capable of exerting a bystander effect [48]. All of these characteristics give T-DXd high stability in plasma and strong antitumor activity across a wide number of HER2+ tumor types [47], including HER2 low tumors or those with heterogeneous expression of HER2 [48]. The antitumoral action of T-DXd on HER2 low tumors has recently been supported by data from the DAISY trial [49].

T-DXd was approved by the FDA for the treatment of HER2+ advanced breast cancer patients who have received at least two prior lines of anti-HER2 therapies [50]. The multicenter single-arm phase II study DESTINY-Breast01 enrolled 184 patients with HER2+ metastatic breast cancer who had been treated with T-DM1 to define T-DXd pharmacokinetics and doses. The study also included patients who suffered tumor progression to T-DM1 or discontinued T-DM1 due to toxicities. The response rate at a dose of 5.4 mg/kg was 60.9% with a confidence interval of 53.4–68.0 and a median duration of the response of 14.8 months [50]. The most common toxicities noted were myelosuppression and nausea, which were associated with interstitial lung disease in 15.2% of patients in grades 1–2, but in 2.7% of patients this toxicity was fatal [51].

Currently, T-DXd is under investigation in over 40 active phase I–III clinical trials registered on ClinicalTrials.gov and comprising different solid organ malignancies, including HER2+ gastric cancer, non-small cell lung cancer, and urothelial or colorectal carcinoma. Its action is also being tested in different breast cancer settings including, among others, residual invasive breast cancer following neoadjuvant therapy (NCT04622319, phase III), alone or in combination with the standard of care in first line therapy (NCT04784715, phase III), in combination with other new anti-HER2 therapies such as tucatinib (NCT04539938, phase II; NCT04538742, phases I/II; and NCT05091528, phases I/II); in HER2 low breast cancer (NCT04494425, phase III; NCT04553770, phase II; NCT03734029, phase III; and NCT04556773, phase I); or in combination with the immunotherapeutic agents nivolumab or pembrolizumab (NCT03523572, phase I and NCT04042701, phase I). In fact, it has been reported that in animal models, T-DXd is able to induce antitumor immunity in combination with anti-CTLA-4 antibodies [52].

Interestingly, T-DXd may also be effective in the context of brain metastases derived from advanced breast cancer. These types of lesions are frequent in advanced settings of the disease and their treatment options are very limited. The DESTINY-Breast01 trial (NCT03248492) demonstrated strong clinical activity of T-DXd, both on HER2+ metastatic breast cancer and brain metastases [53]. This scenario is now being evaluated and validated in several clinical trials (NCT04739761, phase III; NCT04752059, phase II; and NCT04420598, phase II).

### 4.2. Other HER2-Directed ADCs in Clinical Development

#### 4.2.1. Trastuzumab Duocarmycin–SYD985

SYD985 is an ADC in which trastuzumab is linked to the potent DNA alkylating agent seco-duocarmycin-hydroxybenzamide-azaindole (seco-DUBA), an alkaloid isolated from *Streptomyces* [54,55]. Duocarmycins bind to the minor groove of DNA and irreversibly alkylate adenine at the N3 position, disrupting nucleic acid structure and leading to cell death [56,57]. SYD985 contains a linker cleavable by proteases at a valine-citrulline dipeptide (Figure 1). Interestingly, SYD985 has been shown to be able to overcome resistance to T-DM1 in vitro using both cellular and animal models [58]. SYD985 has been granted fast track designation status by the FDA for metastatic breast cancer [54,59]. Several trials are testing its action on different malignancies and stages, such as advanced or metastatic breast cancer in the TULIP trial (NCT03262935, phase III); HER2 low tumors (NCT04602117, phases I/Ib); the safety, pharmacokinetics, and efficacy on locally advanced or metastatic solid tumors (NCT02277717); recurrent, advanced, or metastatic endometrial carcinoma (NCT04205630, phase II); or in combination with niraparib in patients with solid tumors (NCT04235101, phase I) (Table 1).

#### 4.2.2. ARX788

ARX788 is based in a humanized anti-HER2 antibody that is modified with a nonnatural amino acid (*p*-acetyl-phenylalanine, *p*AcF). The latter binds, through a non-cleavable amberstatin drug-linker (AS269), to the N-terminus of MMAF [60]. The resulting ADC is highly homogeneous with a DAR of 1.9. Preclinical data indicate that it exhibits elevated serum stability in animal models with a relatively long half-life (12.5 days) [61]. This compound may overcome resistance to other anti-HER2 therapies, such as T-DM1 [61,62]. ARX788 is currently being tested in several phase II clinical trials in advanced metastatic HER2+ breast cancer resistant to other anti-HER2 therapies (NCT05018702, NCT048299604); in combination with the small HER2 tyrosine kinase inhibitor pyrotinib (NCT04983121); in HER2-low breast cancer (NCT05018676, phase II); and in other HER2+ tumors (NCT05041972, NCT03255070, and NCT02512237) (Table 1). Based on all the preclinical and clinical data, and similarly to SYD985, ARX788, has been granted a fast-track designation by the FDA (January 2021) in patients with advanced or metastatic breast cancer who have previously received one or more HER2-targeted regimens in the metastatic setting, pending confirmatory studies.

#### 4.2.3. RC48-ADC

Disitamab vedotin (RC48-ADC) is an anti-HER2 humanized antibody conjugated to MMAE via the cleavable linker maleimidocaproyl-valyl-citrullinyl-*p*-aminobenzyloxycarbonyl (mc-val-cit-PABC), with a typical DAR of 4. Several preclinical studies have demonstrated the potent antitumoral action of this conjugate, both in vitro and in vivo [63,64,65]. In addition, promising results have been obtained in the preclinical setting using animal models, when combining this therapy with immune checkpoint inhibitors [66]. Based on these promising preclinical data, several clinical studies are verifying the beneficial effects of this ADC in different settings [67,68], including phase I/II and phase III trials and in advanced solid tumors, including breast cancer (NCT02881138, phase I; NCT02881190, phase I; NCT03052634, phase I/II; NCT04400695, phase III; and NCT03500380, phases II/III), non-small cell lung cancer (NCT04311034, phases I/II), gastric tumors (NCT04714190, phase III and NCT03556345, phase II), urothelic/bladder tumors (NTC05016973, phase II; NCT04879329, phase II; NCT04264936, phases I/II; NCT03809013, phase II; NCT03507166, phase II; and NCT04073602, phase II), gynecological tumors (NCT04965519, phase II), and biliary tract malignancies (NCT04329429, phase II). It is being tested as monotherapy or combined with other anti-HER2 therapies, conventional chemotherapeutic agents, or immune checkpoint inhibitors (NTC04280341 and NCT04264936). Interestingly, some of these trials are aimed at investigating the effect of this ADC in HER2-low tumors, an entity that so far cannot benefit from the anti-HER2 therapies available to date (NCT04400695).

#### 4.2.4. ALT-P7

In this ADC, two molecules of MMAE are site-specifically conjugated to a cysteine-containing peptide motif of a trastuzumab variant, and its efficacy is being tested in a phase I clinical trial on metastatic breast cancer (NCT03281824) [69].

#### 4.2.5. MRG002

This ADC also links a humanized anti-HER2 IgG1 to MMAE through a protease cleavable valine-citruline linker, with an average DAR of 3.8. Preclinical studies indicate a favorable toxicity profile and potent antitumor activities in breast and gastric PDX models [70]. Its clinical activity is being investigated in an ongoing phase I study for safety, tolerability, and pharmacokinetics in breast and gastric cancer (CTR20181778) [71]. In addition, several studies are recruiting patients to test this compound in different clinical settings, including gastric, urothelial or biliary tract HER2+ cancer, and HER2 low breast cancer, among others (NCT04924699, NCT04941339, NCT04839510, NCT04837508, NCT04742153, and NCT04492488).

#### 4.2.6. A166

This agent uses the MMAF-derivative duostatin-5 as the warhead. Its action is being tested in a phase I/II clinical trial in monotherapy on several HER2+ solid tumors (NCT03602079) [72].

#### 4.2.7. PF-06804103

This ADC is comprised of a humanized IgG1 anti-HER2 antibody site-specifically linked to auristatin 0101 through a protease cleavable linker [73,74]. The action of this compound is now being analyzed in a phase I clinical trial (NCT03284723) [75].

#### 4.2.8. MEDI4276

MEDI4276 is a biparatopic multiepitope ADC that uses the scFV of trastuzumab and the payload MMETA, a derivative of tubulysin also known as AZ13599185, bound through site specific conjugation to two engineered cysteine residues on the heavy chain of the antibody via a maleimidocaproyl protease cleavable linker [76,77]. The characteristics and safety of this preparation are currently being evaluated in a phase I/II trial on HER2 expressing advanced solid tumors (NCT02576548), including breast and gastric cancers. First results from this study point to the clinical efficacy of this agent, but with high toxicities [78].

#### 4.2.9. BDC-1001

BDC-1001 is a novel immune-stimulating antibody conjugate consisting of a trastuzumab biosimilar chemically conjugated to a toll-like receptor (TLR) 7/8 agonist to induce immune-stimulation at the tumor site. This agent is being tested in advanced HER2+ solid tumors in a phase I/II trial (NCT04278144).

#### 4.2.10. SBT6050

Similarly, SBT6050, a therapeutic agent comprised of a potent TLR8 agonist bound to a HER2 IgG, has been developed and its action is being evaluated in HER2+ solid tumors alone or in combination with other anti-HER2 or immune therapies (NCT05091528, NCT04460456).

#### 4.2.11. FS-1502 (LCB14-0110)

FS-1502 is a novel prenyl transferase-mediated, site-specific ADC that uses MMAF as the payload, bound to trastuzumab through a beta-glucuronide linker that confers to the final molecule high stability and good pharmacokinetic profile [79]. Its action in vivo is being tested in the phase I clinical trial NCT03944499.

#### 4.2.12. ZW49

In ZW49, a biparatopic anti-HER2 IgG is bound to auristatin through a protease-cleavable linker [80]. Its action is being tested on different HER2+ tumors (NCT03821233) [81].

#### 4.2.13. BAT8001

This ADC incorporates the maytansinoid payload batansine to a humanized anti-HER2 IgG1 through a non-cleavable linker. NCT04189211 and NCT04151329 phase I/II clinical trials are analyzing its action on metastatic breast cancer, with promising results [82]. In addition, a phase III trial has been launched to compare its action to that of lapatinib plus capecitabine (NCT04185649).

#### 4.2.14. GQ1001

GQ1001-HER2 is an ADC in which trastuzumab has been linked to the warhead DM1 through an intelligent ligase dependent conjugation (iLDC) system, and whose action is being evaluated in the phase I clinical trial NCT04450732, in the context of advanced solid tumors.

#### 4.2.15. DHES0815A–RG6148

DHES0815A–RG6148 consists of an anti-HER2 mAb linked to the DNA minor groove crosslinking agent pyrrolo(2,1-c)(1,4)benzodiazepine monoamide (PDB-MA). Preclinical in vitro and in vivo data have demonstrated high antitumoral activity [83]. The safety, tolerability, and pharmaco-kinetic properties of this ADC are being tested in a phase I trial on HER2+ breast cancer patients for whom established treatments have proven ineffective, intolerable, or unavailable (NCT03451162).

Several other ADCs targeting HER2 but with undisclosed payloads are under different degrees of clinical development (Table 1).

## 5. Mechanisms of Resistance

**Figure 3:**
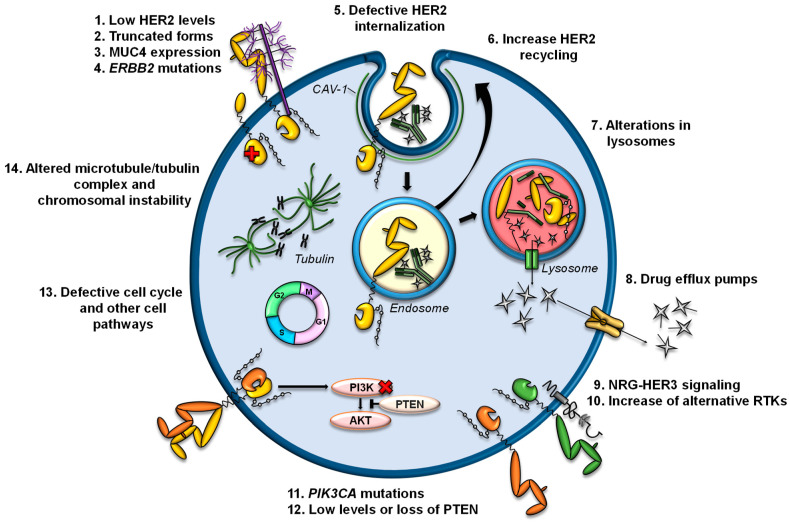
Potential mechanisms of resistance to anti-HER2 ADCs. The mechanisms of resistance to anti-HER2 ADCs are numbered and illustrated. Detailed descriptions are presented in the text.

Several mechanisms of resistance to trastuzumab or T-DM1 have been described (Figure 3). Currently, there are no described mechanisms of resistance to T-DXd, but it is expected that some mechanisms described for trastuzumab and/or T-DM1 can be extrapolated to T-DXd.

### 5.1. Alterations in HER2

T-DM1 and T-DXd are ADCs directed against HER2; therefore, the decrease in the levels of HER2 or structural alterations in this receptor are possible causes of resistance [84,85]. In cell lines and in patient samples, dual treatment with trastuzumab and pertuzumab reduces HER2 levels, decreasing the efficacy of T-DM1 as a second-line treatment [86]. A recent study affirms the need to retest HER2 status in metastatic HER2+ breast cancer after treatment, due to the expression changes observed in HER2 [87]. In that study, patients with loss of HER2 showed worse responses to T-DM1 and inferior OS. Other previous studies have also shown discrepancies (both loss and gain) in HER2 expression levels between primary and metastatic breast cancer lesions, with HER2 loss being more frequent, as well as in samples pre- and post-treatment with anti-HER2 therapies [88,89,90,91,92].

In the KRISTINE trial, a subgroup of 15 patients treated with T-DM1 and pertuzumab who had experienced locoregional progression before surgery showed high heterogeneity in HER2 expression, which may have contributed to the worse clinical outcomes observed in T-DM1 treatment [93]. This phenomenon has also been observed in HER2+ gastric cancer, in which it was reported that a large segment of the patients lost their positivity for HER2 status, and their response to T-DM1 decreased [94]. On the other hand, loss of *ERBB2* amplification was observed in circulating tumor DNA in patients with primary resistance to T-DM1 [95]. In several cell lines generated in vitro with acquired resistance to T-DM1, a decrease in HER2 expression has been observed compared to parental cells [58,62,96,97,98,99,100].

In addition to loss of HER2, the expression of truncated forms of HER2 can also be responsible for resistance to trastuzumab or T-DM1. For example, p95HER2 generates resistance to trastuzumab by lacking subdomain IV, to which trastuzumab binds [101]. The expression of MUC4, a membrane-associated mucin, is also related to resistance to trastuzumab by partially masking HER2 and preventing its binding [102].

A splice variant of *ERBB2* that removes exon 16 in the extracellular domain of HER2 (d16HER2) has been described in breast cancers and HER2+ cell lines [103,104]. Cell lines expressing d16HER2 were initially resistant to trastuzumab (although the trastuzumab epitope was conserved). It has also been reported recently that the d16HER2 isoform confers resistance to T-DM1 in vitro, due to the low internalization of d16HER2 [105]. However, other studies conducted in vivo, with data and samples from HER2+ breast cancer and gastrointestinal patients, indicate that tumors expressing the d16HER2 isoform are sensitive to trastuzumab [106,107,108,109]. Despite these controversies regarding d16HER2, there is no clinical evidence associating d16HER2 with resistance to trastuzumab and/or ADCs targeting HER2 [110].

On the other hand, intracellular mutations in *ERBB2*, mainly in the kinase domain, which have been associated with resistance to trastuzumab [111,112], could also result in resistance to T-DM1 and T-DXd.

### 5.2. Alterations in the Internalization of HER2

Once anti-HER2 ADCs bind to HER2, the complex is internalized into endosomes, a process that can be promoted by endophillin A2 protein [113]. In fact, it has been observed that the silencing of this protein in HER2+ cells reduces HER2 internalization and decreases the response to trastuzumab and T-DM1 [113]. The processes of ubiquitination and trafficking of HER2 to lysosomes can be altered by its constitutive association with the chaperone HSP90 and, therefore, alter the efficacy of HER2-targeted therapies. In fact, 17-AAG-mediated inhibition of HSP90 in combination with trastuzumab enhances the endocytic uptake of HER2 into lysosomes and its degradation. Furthermore, the combination of 17-AAG and trastuzumab has synergistic antiproliferative and apoptotic effects, specifically on HER2+ breast cancer cells [114].

On the other hand, higher recycling of HER2-containing endosomes to the plasma membrane could decrease the arrival of T-DM1 to the lysosome. HER2 is characterized by its rapid recycling, and this process has been observed after the binding of trastuzumab [115]. In a preclinical model of acquired resistance to T-DM1, a greater recycling of T-DM1-HER2 complexes and a reduced intracellular release of DM1 have been observed. However, this model also has lower HER2 levels than the parental line, which also contributes to the resistance phenotype [96].

A recently described mechanism of resistance to T-DM1 is the internalization of T-DM1 through caveolae composed of CAV1, a situation that limits its arrival to lysosomes. In that preclinical model, an overexpression of CAV1 was observed in a cell line with acquired resistance to T-DM1. Furthermore, the colocalization of T-DM1 and CAV1 correlated with a decreased response to T-DM1 in a panel of HER2+ cells. On the other hand, the silencing of CAV1 was not sufficient to re-sensitize the T-DM1-resistant line [98]. In fact, other authors have linked CAV1-dependent internalization with greater sensitivity to T-DM1 [116,117].

### 5.3. Alterations in Lysosomes

The arrival of anti-HER2 ADCs to lysosomes and the processing by lysosomal enzymes is essential for the release of payloads bound to the antibody. Lysosomes are acidic compartments, and their proteolytic capacity depends on the vacuolar proton pump H+-ATPase (V-ATPase) that regulates lysosomal pH. Since lysosomal membranes are impermeable to charged catabolites, Lys-MCC-DM1 requires transport mechanisms from the lysosome to the cytosol. A recent report indicated that an increase in lysosomal pH, as well as a reduction in the activity of lysosomal enzymes, are causes of resistance to T-DM1 [118]. In another model of resistance to T-DM1, a decrease in lysosomal acidification was also reported. In this case, a reduction in intracellular concentrations of Lys-MCC-DM1 was observed because of an aberrant activity of V-ATPase in the lysosomes of resistant cells that decreased the catabolism of T-DM1 and the production of the active catabolite [119].

On the other hand, it has been reported that SLC46A3 is a lysosomal membrane protein that mediates the transport of Lys-MCC-DM1 from the lysosomes to the cell cytoplasm. Loss of *SLC46A3* expression has been observed in several models with acquired resistance to T-DM1, and its lentiviral expression restores sensitivity to T-DM1 [120,121,122].

### 5.4. Increased Expression and Activity of Plasma Membrane Drug Efflux Pumps

Increased expression and/or activity of efflux pumps has been a widely studied mechanism in chemotherapy resistance [123,124]. Regarding T-DM1, it has been reported that some pumps of the ABC family can eject the compound Lys-MCC-DM1 to the extracellular medium, preventing it from binding to tubulin. Specifically, it has been reported that alterations in the expression of ABCB1/MDR1/P-gp, ABCC1/MRP1, ABCC2, and ABCG2/BCRP/MXR/ABCP lead to resistance to T-DM1 in preclinical models. In these studies, inhibition of transporter activity restored sensitivity to T-DM1 [96,120,125,126,127].

### 5.5. Increased Production of Ligands and Activation of Alternative RTKs

In an in vitro study, the addition of the HER3 ligand neuregulin (NRG) inhibited the action of T-DM1 in some HER2+ cell lines [128], due to the consequent dimerization of HER2/HER3 and the activation of the PI3K/AKT pathway. In fact, this effect was reversed by adding pertuzumab, which inhibits this dimerization. These results suggest that ligands such as NRG can affect sensitivity to T-DM1 and therefore be a possible biomarker of refractoriness to T-DM1. A recent study showed that an increase in exogenous NRG1 attenuates the cytotoxic effect of T-DM1 in BT474 cells cultured in 3D. That study also reported that *NRG1* mRNA levels positively correlated with an increase in T-DM1 IC_50_ in a panel of HER2+ cell lines [100]. In fact, in HER2+ breast cancer cell lines with secondary resistance to T-DM1, a higher expression and activation of HER3 and expression of its ligand NRG1 was observed. On the other hand, a significant increase in *NRG1* transcripts was observed in paired tumor samples that progressed after anti-HER2 therapy, including T-DM1 [100]. In this study, a reduction in HER2 protein levels (in 63.6% of the paired samples), and even loss of *ERBB2* amplification, were also observed after treatment.

Two independent studies have reported that in their models with acquired resistance to T-DM1 and primary resistance to trastuzumab, a decrease in HER2 levels has been accompanied by an increase in EGFR levels [99,120]. However, EGFR silencing was not sufficient to reverse the resistant phenotype [120]. Endo et al. observed that increased EGFR levels led to increased amounts of integrins (α5β1 and αVβ3), resulting in augmented motility and invasion of resistant cells.

### 5.6. Alterations in Proteins Involved in Signaling Pathways

Sabbaghi et al. reported that in HER2+ breast cancer cell lines sensitive to T-DM1, treatment with that ADC caused an increase in cyclin B1 and arrest in the G_2_/M phase of the cell cycle, a process that triggers the mitotic catastrophe phenotype characteristic of T-DM1 treatment. However, this phenomenon was not observed in cells resistant to T-DM1, where the accumulation of cyclin B1 did not occur. In that study, the silencing of cyclin B1 in the parental lines generated resistance to T-DM1 and the increase in cyclin B1 levels partially sensitized the resistant cell lines [97]. It should be noted that they also observed that in a cohort of 18 HER2+ breast cancer explants, the effect of T-DM1 paralleled the accumulation of cyclin B1.

In another study, an increase in the expression of a mitotic kinase, polo-like kinase 1 (PLK1), has been observed in models resistant to T-DM1 compared to parental cell lines. Inhibition of PLK1, both at the genomic and pharmacological level with volasertib, reversed resistance to T-DM1 in preclinical models [129].

In cells with acquired resistance to T-DM1, it has been reported that decreased levels of PTEN contribute to the resistance phenotype. In fact, the decrease in PTEN levels in the parental line reduced the action of T-DM1 and trastuzumab. In addition, a PI3K inhibitor reversed resistance to T-DM1 caused by loss of PTEN [120]. In fact, the loss and/or decrease of PTEN levels have been related to resistance to trastuzumab [130,131,132]. In general, deletions in *PTEN* and activating mutations in the gene that encodes the catalytic subunit of PI3K (*PIK3CA*) cause the constitutive activation of PI3K/AKT pathway, which relates to resistance to therapies against HER receptors [3,133].

In T-DM1-resistant esophageal carcinoma cells, alterations in the expression of genes involved in cell adhesion and prostaglandin signaling have been observed [134]. These changes promoted alterations in cell morphology, as well as increased migration and survival. However, the specific mechanism by which these expression changes led to resistance to T-DM1 remains to be elucidated.

Activation of STAT3 mediated by overexpression of LIFR has recently been reported to confer resistance to T-DM1. This activation leads to the expression and secretion of factors that induce resistance. STAT3 inhibition re-sensitizes cells to the action of T-DM1, both in vitro and in vivo [135]. On the other hand, increased expression of ROR1 and an increase in cancer stem cells have been related to resistance to T-DM1 [136].

Recently, an increase in resistance to anti-tubulin agents was observed in a HER2+ breast cancer line with acquired resistance to T-DM1. These cells had lower levels of polymerized tubulin and βIII tubulin and increased baseline aneuploidy. However, the silencing of tubulin βIII in the parental line was not sufficient to generate resistance to T-DM1. The cause of resistance is likely to be multifactorial, including modifications of the microtubule/tubulin complex and chromosomal instability [137].

## 6. Treatment Options in the Anti-HER2 ADC Resistance Scenario

### 6.1. Acting on Payload Extrusion

Some cancer cells develop cross-resistance to a variety of unrelated cytotoxic drugs, a phenomenon known as multidrug resistance (MDR) [124]. ABCB1/MDR1/P-gp is an ABC transporter implicated in MDR in several ADC resistance models [120,138,139,140,141,142]. This protein is a membrane glycoprotein that actively pumps cytotoxic agents out from cells, avoiding intracellular drug accumulation [143,144]. Kovtun et al. overcame MDR1-mediated resistance with a modified linker [145]. They attached DM1 to an antibody using the maleimidyl-based hydrophilic linker PEG_4_Mal. The proteolysis of such conjugates in cancer cells resulted in the release of Lys-PEG_4_Mal-DM1 instead of Lys-MCC-DM1. The former is a poor substrate of MDR1, resulting in potency in killing MDR1-expressing cells [145]. MDR1-mediated resistance could also be overcome by combination strategies using inhibitors of MDR transporters. Nevertheless, these inhibitors have limited clinical application because of the role of ABC transporters in xenobiotic elimination and their expression in non-tumoral cells [146].

### 6.2. Redirecting HER2 Internalization and Recycling

Another strategy for overcoming anti-HER2 ADCs’ resistance and increasing their efficacy is to modulate HER2 recycling [115]. Geldanamycin, an inhibitor of HSP90, decreases HER2 recycling and induces localization of HER2 in intracellular vesicles improving degradation of HER2 [115,147]. Indeed, as mentioned before, 17-AAG, another HSP90 inhibitor, in combination with trastuzumab, enhanced HER2 ubiquitination and its downregulation from the cell surface, and also promoted HER2 lysosomal degradation. In addition, the combined therapy potentiated cytotoxicity in HER2+ breast cancer [114]. For that reason, it can be speculated that the combination of an anti-HER2 ADC followed by HSP90 inhibitors may inhibit recycling of ADC-HER2 complexes and direct such complexes for lysosomal degradation.

### 6.3. Advances in Anti-HER2 ADC Development in Preclinical Stage

In addition to the anti-HER2 ADCs under clinical development that have been discussed above, several anti-HER2 ADCs that include cleavable linkers, new conjugation techniques, specific conjugation sites, increased DAR, and/or new cytotoxic agents that possess bystander cytotoxic effect are currently being developed. Those changes improve the stability and efficacy of ADCs compared to T-DM1, and therefore improve their activity in tumors with heterogeneous HER2 expression and/or resistance to T-DM1 [125,148,149,150].

The current improvements in anti-HER2 ADCs are mainly based in stable DARs with new conjugation technologies and linker chemistry [151]. T-PNU is a new HER2-targeting ADC in which trastuzumab is coupled to a derivative of the highly potent anthracycline PNU-159682 via a non-cleavable peptide linker, by sortase-mediated antibody conjugation (SMAC) technology [148]. Another strategy used to improve anti-HER2 ADCs is the use of two payloads to construct the ADC. Following this strategy, an anti-HER2 ADC containing two different cytotoxic agents (MMAF and MMAE) has been generated with effectiveness in HER2+ breast cancer tumors [125]. Yamazaki and colleagues conjugated both payloads to an anti-HER2 mAb with an N297A mutation by chemoenzymatic conjugation. This approach allowed the simultaneous delivery of two payloads with different features and was able to combat breast cancer HER2 heterogeneity and drug resistance to T-DM1 [125].

Tra-Exa-PSAR10 is a novel ADC anti-HER2 generated by a specific technology called hydrophilic monodisperse polysarcosine (PSAR) drug-linker platform (PSARlink) [149]. With this technology, Coniln and colleagues conjugated trastuzumab to the topoisomerase I inhibitor payload exatecan, achieving a DAR of 8. Tra-Exa-PSAR10 showed higher bystander killing effect than T-DXd and overcame resistance to T-DM1 in HER2+ breast and gastric cancer models.

Robinson and colleagues reported the use of pyridazinediones to selectively re-bridge the native solvent-accessible interstrand disulfide bonds in trastuzumab with MMAE. This method of conjugation generated serum-stable ADCs with a controlled DAR of 4 [150]. Moreover, they reported that this generated MMAE-trastuzumab ADC is potent, selective, and efficacious against HER2+ breast cancer models.

### 6.4. Therapies against HER3

Overexpression of HER3 is often associated with overexpression of HER2, playing an important role as co-receptor in HER2+ breast cancer [152,153]. Furthermore, breast tumors often show co-expression and positive correlation between HER2 and HER3 levels [154,155].

Elgemtumab or LJM716 is a fully human IgG1 mAb against an epitope located between domains II and IV of the ectodomain of HER3. Treatment with this antibody resensitized cellular models with acquired resistance to T-DM1 to the action of that drug [100].

EV20/MMAF, an ADC against HER3 composed by the mAb EV20 and the payload MMAF [156], has recently demonstrated antitumoral activity in T-DM1-resistant models [157]. In addition, this ADC showed antiproliferative activity in other anti-HER2 therapy resistant settings, including models resistant to trastuzumab, lapatinib, and neratinib [157].

MCLA-128 is a bispecific IgG1 targeting HER2 and HER3. MCLA-128 has a “dock and block” mechanism that blocks the HER2/HER3 heterodimerization. The mechanism of action of this bispecific antibody also includes enhanced ADCC. This antibody has demonstrated its potency in models resistant to T-DM1 [158]. It has also been effective in patients with *NRG1* fusions [159]. MCLA-128 is currently being tested in phase I/II clinical trials, which report that MCLA-128 is very well tolerated and safe (NCT03321981, NCT02912949, and NCT04100694).

### 6.5. Pan-HER and TKI Thrapies

Another strategy to overcome anti-HER2 therapy resistance and avoid pathway compensation between ErbB/HER-family receptors is the use of pan-HER antibody mixtures directed against EGFR, HER2, and HER3. The effectiveness of such a strategy has been described in tumors resistant to trastuzumab and T-DM1, demonstrating that such a combination reduces the appearance of relapses compared to other anti-HER2 therapies in clinical use [100,160]. Pan-HER (also called Sym013) is a mixture of 6 mAbs, comprising three pairs of synergistic mAbs, each targeting EGFR, HER2, and HER3 [160,161]. This mixture promotes degradation of the receptors and prevents compensatory receptor up-regulation, induces ADCC and complement-dependent cytotoxicity, affects the presence of ligands, and therefore inhibits activation of the PI3K/AKT and MAPK/ERK pathways. Sym013 effectively inhibited growth of HER2+ breast cancer models in vitro and in vivo, including models resistant to HER2-targeted therapeutics such as trastuzumab and T-DM1 [100]. The effect of this compound has been evaluated in different advanced epithelial malignancies (NCT02906670).

A novel HER2-selective irreversible TKI (TAS0728) has recently been described as effective in tumors with acquired resistance to trastuzumab and pertuzumab and to T-DM1. Treatment with TAS0728 resulted in a significant antitumor effect due to HER2-HER3 signal inhibition [162]. Such TKI is currently in phases I/II clinical evaluation.

Neratinib (Nerlynx^®^) is an irreversible pan-HER TKI [163,164,165]. Recently, it has been reported that the cotreatment of T-DM1 with irreversible pan-HER TKIs (such as neratinib and afatinib) enhances the ubiquitination of HER2 and its internalization, and consequently the internalization of T-DM1 and its efficacy. In one patient who showed progression after treatment with T-DM1, the addition of neratinib improved the response to T-DM1 [166]. In 2019, a clinical trial was published showing the efficacy and good tolerability of T-DM1 plus neratinib in patients with metastatic HER2+ breast cancer that progressed to trastuzumab, pertuzumab, and taxane [167]. These results underscore the relevance of the double blockade strategy for HER2+ tumors, based on the combination of an antibody acting on the ectodomain plus a TKI.

### 6.6. Combination of ADCs with Other Therapies

Encouraging data suggest that ADCs can enhance antitumor immune response and improve clinical outcomes. Müller et al. reported that T-DM1 treatment combined with anti-CTLA-4/anti-PD-1 was more effective than monotherapy, because the combination triggered innate and adaptive immunity [168]. In fact, antitumor immunity was seen in response to T-DM1, but not to trastuzumab, suggesting that DM1 might be responsible for triggering modulatory properties. In line with this observation, the anthracycline payload of T-PNU induced immunogenic cell death [148]. Indeed, the anticancer activity of T-PNU is related to the adaptive immune system. D’Amico et al. demonstrated that the combination of the T-PNU and anti-PD-1 blocking antibodies significantly enhanced antitumor activity, suggesting that stimulation of immunogenic cell death sensitizes to immunotherapy [169]. It has been shown that U3-1402, an ADC targeting HER3, improved the function and infiltration of innate and adaptive immune cells, which subsequently sensitized the tumor to immunotherapy. Accordingly, the combination of this ADC and an anti-PD-1 antibody significantly inhibited tumor growth, even in mice refractory to anti-PD-1 therapy [170]. Currently, combinations of atezolizumab (PD-L1 inhibitor) or pembrolizumab (PD-1 inhibitor) with T-DM1 are under evaluation in several clinicals trials with promising results [171,172].

Inhibition of the antiapoptotic proteins BCL-2 and BCL-X_L_ via navitoclax/ABT-263 significantly enhanced the cytotoxicity of T-DM1 in models derived from advanced and pretreated metastatic breast tumors. Those models, which were highly responsive to the combined therapy, lacked *ERBB2* amplification and had low HER2 expression, suggesting that a BCL-2/X_L_ blockade could increase sensitivity of tumors with low HER2 protein levels [173].

## 7. Conclusions

To date, there are twelve FDA-approved ADCs; two of them, T-DM1 and T-DXd, are indicated for HER2+ breast cancer, with the latter also indicated in patients with HER2+ gastric cancer. Despite the unquestionable clinical benefit obtained in patients with HER2+ tumors, a number of these patients develop resistance to T-DM1. In addition, some HER2-positive cancers are primarily nonsensitive to T-DM1. Therefore, strategies to overcome such resistances are required.

The development of T-DXd represents one of the first strategies in overcoming resistance to T-DM1 and exemplifies the value of using different chemotherapeutic agents or coupling chemistries to create ADCs that may be effective under circumstances of resistance to T-DM1. Therefore, optimization of each ADC component (the antibody, the linker, and the payload), the conjugation site, and chemistry can improve the efficacy of novel ADCs against tumors refractory to the ADCs currently used in clinics. In addition, identification of the mechanisms of resistance may allow for the development of treatments to overcome them. In this respect, the use of drugs that show preclinical capability to overcome such resistance represents an important initial step.

## Figures and Tables

**Table 1:** Anti-HER2 ADCs approved for use or in clinical development (in clinical trials).

ADC	Antibody	Payload	Linker	DAR	Status	Clinical Trials and References
T-DM1	Trastuzumab	DM1	Non-cleavable	3.5	Approved	
T-DXd	Trastuzumab	Deruxtecan	Cleavable	6–8	Approved	
SYD985	Trastuzumab	Duocarmycin (seco-DUBA)	Cleavable (valine-citruline)	2.8		NCT03262935, phase III NCT04602117, phase I/Ib NCT02277717, phase I NCT04205630, phase II NCT04235101, phase I
ARX788	Modified trastuzumab	MMAF	Non-cleavable (Amberstatin, AS269)	1.9	CT	NCT05018676, phase II NCT05018702, phase II NCT048299604, phase II NCT04983121, phase II NCT05041972, phase II NCT03255070, phase I NCT02512237, phase I
RC48-ADC	Ig anti-Her2	MMAE	Cleavable (valine-citruline)	4	CT	NCT03052634, phase I/II NCT04400695, phase III NCT03500380, phase II/III NCT04311034, phase I/II NCT04714190, phase III NCT03556345, phase II NTC05016973, phase II NCT04879329, phase II NCT04264936, phase I/II NCT03809013, phase II NCT03507166, phase II NCT04073602, phase II NCT04965519, phase II NCT04329429, phase II NCT04280341, phase I NCT02881138, phase I NCT02881190, phase I
ALT-P7	Trastuzumab variant	MMAE	Cysteine -containing peptide	UD	CT	NCT03281824, phase I
MRG002	IgG anti-HER2	MMAE	Cleavable (valine-citruline)	3.8	CT	CTR20181778, phase I NCT04924699, phase II NCT04941339, phase I NCT04839510, phase II NCT04837508, phase II NCT04742153, phase II NCT04492488, phase I/II
A166	Trastuzumab	MMAF	Cleavable (valine-citruline)	UD	CT	NCT03602079, phase I/II
PF06804103	IgG1 antiHER2	Auristatin 0101	Cleavable (Maleimidocaproyl-valine-citruline)	4	CT	NCT03284723, phase I
MEDI4276	Trastuzumab scFV	MMETA	Protease cleavable	4	CT	NCT02576548, phase I/II
BDC1001	Trastuzumab biosimilar	TLR 7/8 agonist	Non-cleavable	UD	CT	NCT04278144, phase I/II
SBT6050	IgG anti-HER2	TLR8 agonist			CT	NCT05091528, phase I/II NCT04460456, phase I
FS-1502	Trastuzumab	MMAF	Beta-glucuronide	2	CT	NCT03944499, phase I
ZW49	Biparatopic antiHER2 IgG	Auristatin	Cleavable	UD	CT	NCT03821233, phase I
BAT8001	IgG1 antiHER2	Batansine	Non-cleavable	UD	CT	NCT04189211, phase I NCT04151329, phase I/II NCT04185649, phase III
DHES0815A	IgG antiHER2	PBD-MA	Non-cleavable	2	CT	NCT03451162, phase I
GQ1001	Trastuzumab	DM1	UD Intelligent ligase dependent conjugation	UD	CT	NCT04450732, phase I
DP303c	IgG antiHER2	UD			CT	NCT04146610, phase I NCT04828616, phase II NCT04826107, phase II
BB-1701	IgG antiHER2	UD			CT	NCT04257110, phase I
SHR-A1811	IgG antiHER2	UD			CT	NCT04446260, phase I NCT04818333, phase I/II NCT04513223 phase I
B003-101	IgG antiHER2	UD			CT	NCT03953833, phase I

Abbreviations: DAR = drug to antibody ratio; CT = clinical trial; UD = undisclosed; MMAE = monomethyl auristatin E; and MMAF = monomethyl auristatin F.
